# Choosing the best way: how wild common marmosets travel to efficiently exploit resources

**DOI:** 10.1007/s10071-024-01864-8

**Published:** 2024-03-02

**Authors:** Dêverton Plácido Xavier, Filipa Abreu, Antonio Souto, Nicola Schiel

**Affiliations:** 1 Laboratory of Theoretical and Applied Ethology, Department of Biology, Federal Rural University of Pernambuco, Recife, Brazil; 2 Laboratory of Ethology, Department of Zoology, Federal University of Pernambuco, Recife, Brazil

**Keywords:** Heuristic strategy, *Callithrix jacchus*, Spatial navigation, Travel route, Route planning

## Abstract

While foraging, animals have to find potential food sites, remember these sites, and plan the best navigation route. To deal with problems associated with foraging for multiple and patchy resources, primates may employ heuristic strategies to improve foraging success. Until now, no study has attempted to investigate experimentally the use of such strategies by a primate in a context involving foraging in large-scale space. Thus, we carried out an experimental field study that aimed to test if wild common marmosets (*Callithrix jacchus*) employ heuristic strategies to efficiently navigate through multiple feeding sites distributed in a large-scale space. In our experiment, we arranged four feeding platforms in a trapezoid configuration with up to 60 possible routes and observe marmosets’ decisions under two experimental conditions. In experimental condition I, all platforms contained the same amount of food; in experimental condition II, the platforms had different amounts of food. According to the number and arrangement of the platforms, we tested two heuristic strategies: the Nearest Neighbor Rule and the Gravity Rule. Our results revealed that wild common marmosets prefer to use routes consistent with a heuristic strategy more than expected by chance, regardless of food distribution. The findings also demonstrate that common marmosets seem to integrate different factors such as distance and quantity of food across multiple sites distributed over a large-scale space, employing a combination of heuristic strategies to select the most efficient routes available. In summary, our findings confirm our expectations and provide important insights into the spatial cognition of these small neotropical primates.

**Supplementary Information:**

The online version contains supplementary material available at 10.1007/s10071-024-01864-8.

## Introduction

Wild animals are constantly challenged with complex spatial problems when navigating through their environment (Riotte-Lambert and Matthiopoulos 2020; Abreu et al. 2021). While foraging, animals have to find potential food sites, remember such sites, and plan the best navigation route (Cunningham and Janson 2007; Janson 2007; Garber and Porter 2014; Trapanese et al. 2018). Thus, they face a problem similar to the Traveling Salesman Problem (TSP) (Gärling 1989; Teichroeb and Smeltzer 2018), which consists of finding the best route among multiple locations (e.g., feeding sites, resting sites, etc.) and returning to the starting point (Teichroeb and Smeltzer 2018; Trapanese et al. 2018). The TSP poses a combinatorial optimization problem (i.e., a finite number of locations with resources available to explore and discrete decision variables; Hoos 2005; Karimi-Mamaghan et al. 2022), which is difficult to solve, and the problem-solving time increases in proportion to the number of locations to be visited (Janson 2013). However, foraging challenges occur also when animals do not need to return to their starting point (sleeping site). In this case, we refer to the problem as an open-TSP or optimal Hamiltonian path problem (Janson 2013). Due to its complexity, animals are not expected to solve the TSP or even the open-TSP, but they come up with relatively optimal paths through heuristic strategies (Chronicle et al. 2006; Janson 2013) and just apply a general rule for site selection. These heuristic strategies ignore some information about the main problem (e.g., location of all resources, length of all possible routes, etc.) so that decisions can be made faster and more efficiently (Gigerenzer and Gaissmaier 2011).

Heuristic strategies (e.g., gravity rule, nearest neighbor rule, cluster strategy, convex hull strategy, 3-step look ahead rule; for more details see Janson 2013 and Teichroeb 2015) can involve simple decisions, such as the Nearest Neighbor Rule (hereafter NNR): the animal always chooses the route with the feeding site closest to its current position (Teichroeb and Smeltzer 2018), or more complex decisions, like the Gravity Rule (hereafter GR): the animal chooses its routes in accordance to the attractiveness of the destination, considering distance and reward availability (Janson 2013). The NNR is considered less demanding from a cognitive standpoint as it only requires moving between the resources closest to the individual’s current location (Janson 2013; Teichroeb 2015). This strategy allows the animals to factor in only the distance between the sites of interest. The NNR is adopted by several species, including the domestic pigeon, *Columba livia* (Miyata and Fujita 2010; Gibson et al. 2012), the rufous hummingbird, *Selasphorus rufus* (Tello-Ramos et al. 2015), the anubis baboon, *Papio anubis* (Farine et al. 2016), and the vervet monkey, *Chlorocebus pygerythrus* (Teichroeb 2015; Teichroeb and Aguado 2016). On the other hand, the GR requires the animal to follow routes based on the cost-benefit ratio (profitability) between the distance and the reward available at the sites of interest. This means that the desirability of each feeding site would be directly proportional to the available reward and inversely proportional to the distance the animal would need to travel to access the food (Sen and Smith 1995; Janson 2000, 2013).

However, heuristic strategies are not always mutually exclusive (Lihoreau et al. 2011; Teichroeb and Aguado 2016; Reyna-Hurtado et al. 2018). For example, some authors documented that vervet monkeys (*Chlorocebus pygerythrus*) use the GR, the NNR, or cluster strategies (i.e., first, the animal chooses large, clustered feeding sites and then moves to smaller clustered feeding sites; Teichroeb 2015) according to their hierarchical position and the presence or absence of other individuals competing for the same food resource (Teichroeb and Aguado 2016). Furthermore, when making their routing decisions, vervet monkeys take into consideration their handling skill, the composition of the group members and their social relationships, and the distance of certain individuals (Arseneau-Robar et al. 2022). On the other hand, other researchers found that buff-tailed bumblebees (*Bombus terrestris*) adjust their navigation strategy to the presence of more productive flowers and the distance they need to travel to exploit them (Lihoreau et al. 2011). Thus, animals presumably seek to adjust their travel routes to optimize foraging strategies based on the challenges they face.

While many studies aimed to evaluate the use of heuristic strategies during navigation either in large or in small-scale space by different species (e.g., insects: Lihoreau et al. 2010, 2012a; Buatois and Lihoreau 2016; Woodgate et al. 2017; Latty and Trueblood 2020; birds: Miyata and Fujita 2010; Gibson et al. 2012; Tello-Ramos et al. 2015; primates: Garber and Dolins 1996; Cunningham and Janson 2007; Janson 2013; Garber and Dolins 2014; Shaffer 2014; Hopkins 2016; Teichroeb and Aguado 2016; Teichroeb and Smeltzer 2018; Teichroeb and Vining 2019), to the best of our knowledge, no study has attempted to investigate the use of such strategies experimentally by a primate foraging in large-scale space. In this scenario, animals are faced with an additional challenge: potential feeding sites are not visible (Garber and Porter 2014). Navigating in this spatial scale becomes especially interesting as the animal needs to rely on certain cognitive abilities such as spatial memory and episodic memory, due to the uncertainty of the amount and type of food available (Garber and Porter 2014; Abreu et al. 2021).

Although testing primate navigational choices poses a notable challenge, experimental field research offers the opportunity to study animals’ strategies in the complicated context in which they evolved (Janson 2007; Teichroeb 2015; Teichroeb and Smeltzer 2018). Wild common marmosets have proven to be excellent models in these experiments (Caselli et al. 2018; Abreu et al. 2020; De la Fuente et al. 2021). Common marmosets are omnivorous (Souto et al. 2008) and their diet also includes fruits. This is particularly interesting since these resources are stationary, have a patchy distribution in space and their availability varies over time due to the fruiting cycles, which leads these animals to forage in a non-random fashion to minimize the traveled distance and maximize their energy gain (Pyke 1984). Frugivory has been related to both larger brain size (Milton 1981; Barton 2000/2006; DeCasien et al. 2017) and the use of more complex foraging strategies (Milton 1981; Teichroeb and Vining 2019). With that in mind, we conducted an experimental field study that aimed to test if wild common marmosets (*Callithrix jacchus*) use routes consistent with heuristic strategies to efficiently navigate through multiple feeding sites with different amounts of food availability distributed in a large-scale space. Given that these small neotropical primates have an omnivorous diet (Schiel and Souto 2017; Malukiewicz et al. 2021) and published literature has shown that this species possesses spatial cognitive abilities (Abreu et al. 2020, 2021), we expect that wild common marmosets will use routes consistent with heuristic strategies more than expected by chance to efficiently navigate between multiple feeding targets under different scenarios. Additionally, we expect that common marmosets will use the GR strategy more than expected by chance, particularly in scenarios with varying amounts of available food.

## Methods

### Study area and subjects

The study was carried out at the Baracuhy Biological Field Station, located inland in the state of Paraíba, Northeast Brazil (7º31’42’’ S; 36º17’50’’ W). This is a Caatinga area, characterized by a predominance of hyperxerophytic shrubby vegetation (Nascimento and Alves 2008), dominated by small, scattered trees (De La Fuente et al. 2014). The vegetation cover is scarce and sparse with few waterways, and the average annual rainfall is less than 600 mm, with rainfall mostly occurring in a 4-month period, from March to June (Nascimento and Alves 2008; Medeiros et al. 2016).

During the data collection period, the study group consisted of 10 individuals (adults: 3 males / 1 female; subadults: 2 males; juveniles: 2 of unidentified sex; infants: 2 of unidentified sex). The size of the study group is considered representative of the study area, as the average group size for this species in the Caatinga is around 6 animals (Abreu et al. 2016, 2020; Caselli et al. 2018; Garber et al. 2019). Moreover, other field studies on spatial memory have used a single group with a size ranging from 6 to 41 individuals (e.g. Janson 2007; Presotto et al. 2018; Abreu et al. 2021). Since the foraging skills of juveniles and infants are still not fully developed (Missler et al. 1992; Souto et al. 2007; Schiel et al. 2010), we decided to focus our data collection on adult and sub-adult individuals (n *=* 6). Marmosets were identified by natural marks, sex, age (Schiel and Huber 2006; Schiel et al. 2008a, b), or by tags (e.g., collars) from previous studies (De La Fuente et al. 2019).

### Experimental design

In January 2019, preliminary observations were conducted for 10 consecutive days (120 h in total) to determine the area used by the group. Marmosets were followed throughout their activity period (5 a.m. to 5 p.m.) using a 2-min. focal instantaneous sampling technique, which consisted of recording the behavior and geographic location (via Garmin Etrex 10 GPS) of the focal animal every two minutes (Garber and Porter 2014; Abreu et al. 2021) during the whole day. If an individual was out of sight for two consecutive scans (4 min), we moved on to the nearest marmoset on the right (a more detailed description of the observation method is provided below).

**Fig. 1:**
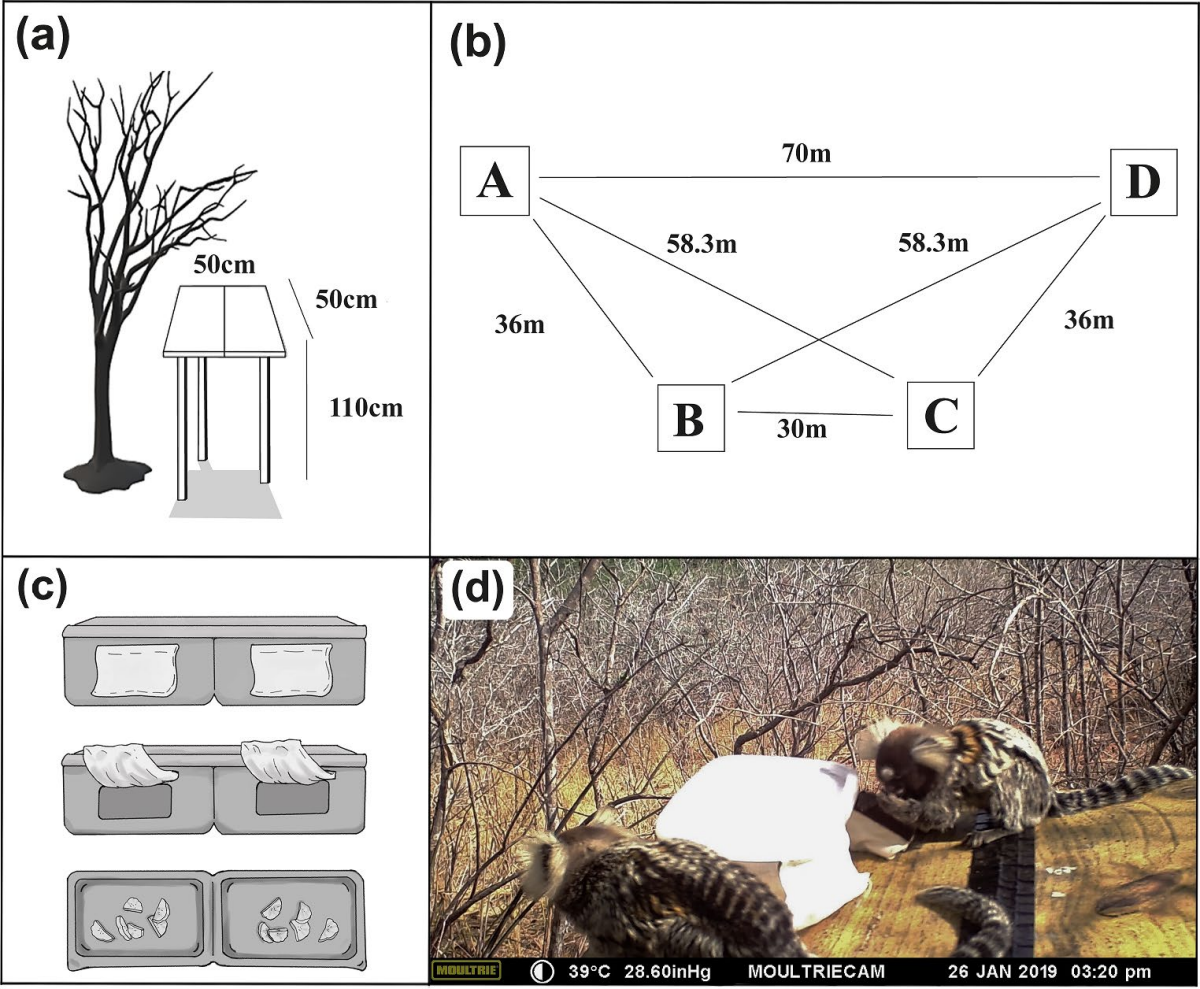
Illustration of the experimental design: (**a**) illustration of a platform next to a tree to provide easy access for the animals; (**b**) layout of the spatial arrangement of the platforms (squares) in the experimental area with their respective distances; (**c**) schematic image of the plastic food container used in the experiment, showing respectively, from top to bottom: its front view with the openings covered by an opaque white fabric, which is lifted to show the openings in dark gray; and its top view without the lid, showing the pieces of banana inside in light gray and the fabric covering the openings in translucent gray; (**d**) two adult male marmosets on top of platform A feeding on pieces of banana from the plastic container

During the preliminary data collection phase, we also chose a location commonly visited by the animals to set up four 50 cm × 50 cm platforms, elevated 110 cm off the ground (Fig. 1a). All platforms were equally easy to access and had the same vegetation cover around them. The platforms were arranged in a trapezoid pattern at the following distances: 70 m (A-D), 30 m (B-C), and 36 m (A-B and C-D) (Fig. 1b) with the objective of testing two different heuristic strategies, namely NNR and GR. These distances were measured using a measuring tape and based on a visibility test performed by the first and second authors. In this test, the authors measured the minimum distance required for a vividly colored object (e.g., a wooden disc painted in red) to no longer be visible at approximately the height of the trees (~ 2 m high), where the animals normally forage. However, it should be noted that this is an average height, used for visibility measurement purposes (e.g., Abreu et al. 2021) and, in the Caatinga, animals may forage in the higher treetops (≥ 3 m), as well as on the ground (Schiel and Souto 2017). On each platform, we placed a plastic container with two compartments (Fig. 1c, d) in which we placed small banana pieces (~ 3 g each) to make it possible for at least two individuals to grab food at the same time (De la Fuente et al. 2019). Each compartment had a front opening providing access to the food. To eliminate the effect of possible visual cues about the amount of food provided in each container, the lids of the containers were covered with opaque paper and the front openings were covered with a small cotton fabric held in place by a Velcro® strap (Fig. 1c, d).

In this preliminary stage, we estimated the amount of food required for all individuals in the group to be satiated through a satiety test. The procedure was conducted to maintain the interest of the animals in the experiment, ensure motivation, and avoid competition for resources during sampling in the experimental phase. Thus, we offered 40 banana pieces (3 g each) inside the plastic containers without the lid on one single platform once a day. After all the individuals ate and moved at least 30 m away from the platforms, we counted the number of pieces consumed. At the end of eight sessions, we calculated the average number of banana pieces consumed by the group. We found that 28 pieces were enough to satiate all group members. To avoid influencing the animals to use a specific route to visit the platforms, each day the food was offered on one single platform.

**Fig. 2:**
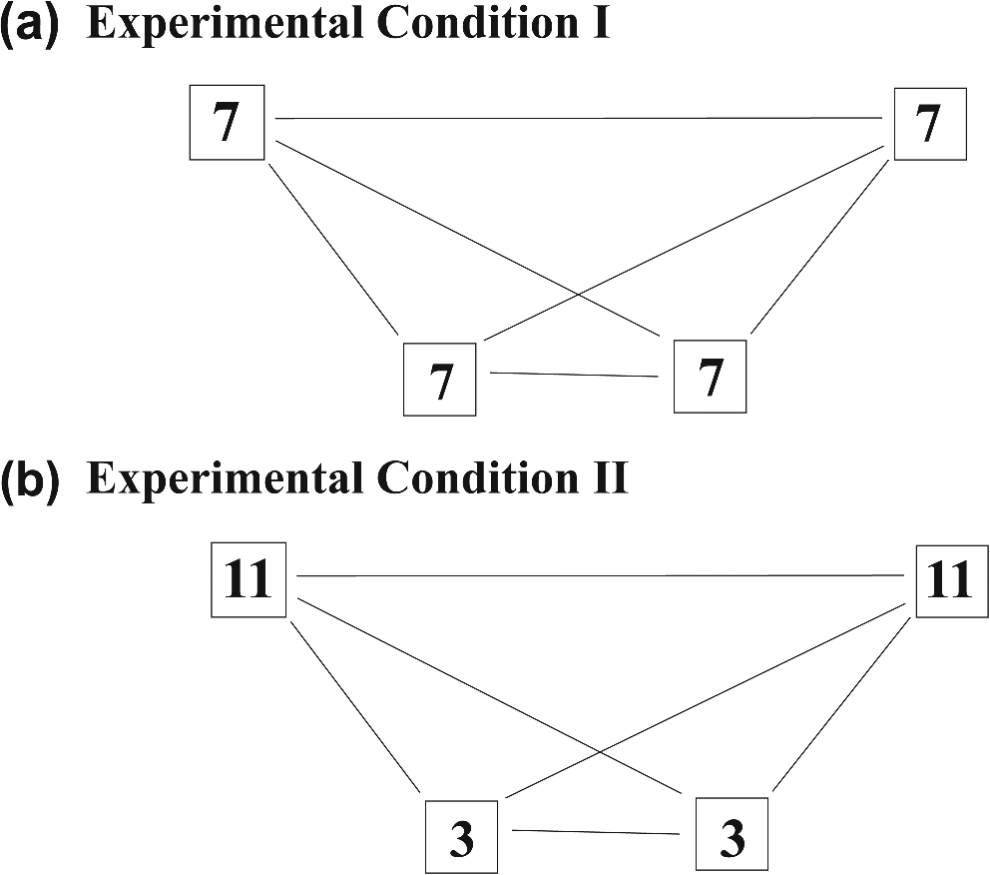
Illustration showing the number of pieces of banana stored in the plastic food containers on each platform during (**a**) experimental condition I and (**b**) experimental condition II

The experiment consisted of two conditions: experimental condition I – same amount of food on each platform (7 pieces of banana); experimental condition II – more food on the platforms at the edge of the arrangement (platforms A and D: 11 pieces of banana) and less food on the platforms closest to each other (platforms B and C: 3 pieces of banana) (Fig. 2). The aim of the two experimental conditions was to exemplify the scenarios that marmosets face in the wild. These two different scenarios are expected to have an influence on the routes chosen by the studied individuals. Since this study investigates the use of spatial memory in route selection by common marmosets, we discarded the first four sessions in each experimental condition, so that the animals could learn the location and amount of food available in each platform (Abreu et al. 2020).

### Data collection

The data were collected between January and March 2019 by the first author, who performed two experimental sessions per day. We observed one animal per day using a 2-min. instantaneous focal sampling technique (Abreu et al. 2021). The focal animals were randomly selected (by drawing) from the studied animals. When the focal animal was lost by the researcher for two or more scans (≥ 4 min.), it was replaced by the nearest adult or subadult individual to the right of the researcher. Subsequently, the observation continued with the newly focal animal, and there was no return to the previous one, even if it came into view again. This method was already used by Abreu et al. (2021) as marmosets form cohesive groups and feed and travel together (Schiel and Huber 2006). For each focal animal, we tried to record approximately the same number of experimental sessions in the morning and afternoon periods. Each experimental session began when the focal animal was within 5 m of any given platform and was considered concluded when the same individual did not visit any platform for 30 min or move and stayed ≥ 30 m away from any of the platforms (for example, if the focal animal visited one platform and then moved on to forage in other locations for more than 30 min, we did not consider this session as valid). By the end of our study, we had a total of 75 valid sessions (an average of 6 sessions per individual in each experimental condition), equivalent to a total of 456 h of direct observations.

Any sequential visit to two or more platforms by the focal individual was counted as a ‘route’ (see Janson 2007). We recorded the order of subsequent visits to the platforms by the focal animal. We only considered it a visit when the animal climbed onto the platform and reached into the container (i.e., when there was evidence of a search for the food items contained therein).

The platforms were stocked with food twice a day (~ 5 a.m. and ~ 12 p.m.), allowing the animals to access the platforms during peak foraging hours (~ 6 a.m. and 2 p.m.: see De La Fuente et al. 2014). To avoid the influence of visual and/or auditory cues on the platform choice, food refills were carried out only when the animals were sufficiently far from any of the platforms (≥ 30 m). After preparing the food for the first session, the observer would head toward the sleeping tree used by the animals (which was identified the previous day) and then start following the focal animal. This was our timeline: stocking the foraging containers @5am; heading to the sleeping tree @5:15am and starting the observation as the focal animal left the sleeping site; starting the first experimental session @between 6am and 10am, once the focal animal reached one of the feeding platforms.

### Data and statistical analysis

Based on the number and arrangement of the platforms, our experiment offered 60 possible routes that were investigated according to two heuristic strategies: the NNR (i.e., the animal chooses an unused feeding site closest to its position; Teichroeb 2015) and the GR (i.e., the animal selects the routes according to the ratio between the amount of the available reward and the distance from the sites of interest; Janson 2013).

**Fig. 3:**
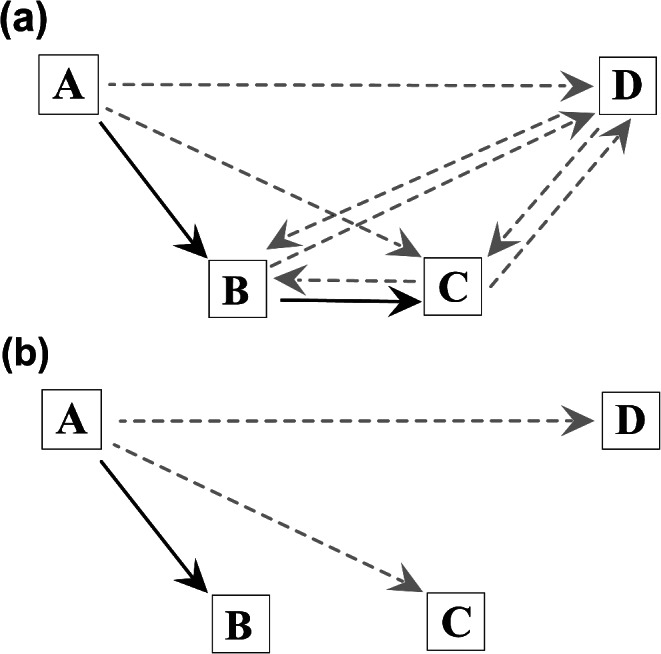
Schematic illustration comparing the heuristic routes (solid arrow in black) and non-heuristic routes (dashed arrow in light gray): (**a**) possible two-step routes from platform A with emphasis on NNR route (“ABC”) in both experimental conditions; (**b**) possible one-step routes from platform A with emphasis on GR route (“AB”) in experimental condition II

To identify NNR routes we considered the starting point and the number of steps required to cover the entire travel distance (i.e., the number of visited platforms to complete the route after the first visit; for example, a route of 3 steps indicates that the animal sequentially moved between all 4 platforms). To do so, we calculated the distance the animals would have to travel between each platform to complete the route (i.e., whether they choose to reach the nearest unused platform or not) since this strategy only factors in the distance. For example, of the six possible two-step routes starting from platform A (ABC, ABD, ACB, ACD, ADB, and ADC), the one classified as an NNR route is ABC, because starting from A, the nearest platform is B, and from B the nearest platform is C (see Fig. 3a).

Similarly, we sought to identify GR routes. For this purpose, we compared the 60 possible routes not only considering the distance between the platforms, but also the amount of food available on each one of them. As in the previous case, we considered the starting platform of each route, and the number of steps required to complete the route. We then calculated the profitability index for all the possible routes in each experimental condition. This index is the ratio between the sum of the amount of food provided on these platforms and the distance required to move across the platforms in straight line segments following each route (for more details see Janson 2007):

$$ {\text{Profitability index}}=\frac{\sum  Food \; quantity}{Distance}$$

Therefore, routes with the same starting point and number of steps were classified as “GR routes”, that is, routes with the highest index of profitability. For example, in experimental condition I, starting from platform A, three 1-step routes were possible (AB, AC, and AD, see Fig. 3b). Among these three 1-step routes, only one (AB) is a GR strategy, as it exhibits the highest profitability index.

Finally, we grouped all routes classified as NNR and GR into a single category (heuristic routes), and all other routes into a second category (other paths, i.e., all possible non-heuristic routes during the experiment; e.g., Teichroeb and Smetzer 2018) (Table 1). Pooling the NNR and GR routes in the same category was necessary due to the overlap of their routes. That is, some routes could be classified as both GR and NNR (Table 1). Because the convex hull strategy (hereafter CH) consisted of only three routes, some of which were also consistent with the GR or NNR strategies and were performed only once in both categories (please see Table 1 for more details), we decided to pool this heuristic strategy into the category “heuristic routes”. In each experimental condition, we counted the number of routes consistent with heuristic strategies used by common marmosets.

**Table 1:** Routes, distance, frequency of use and profitability index in both experimental conditions

Route	Distance (m)	Experimental condition I				Experimental condition II			
		Strategy	F	% used	PI	Strategy	F	% used	PI
AB	36	GR/NNR	1	2.38	0.39	GR/NNR	0	0	0.39**
AC	58.3		0	0	0.24		0	0	0.24
AD	70		0	0	0.20		0	0	0.31
BA	36		2	4.76	0.39	GR	1	3.03	0.39**
BC	30	GR/NNR	5	11.9	0.47**	NNR	2	6.06	0.20
BD	58.3		0	0	0.24		0	0	0.24
CA	58.3		0	0	0.24		0	0	0.24
CB	30	GR/NNR	2	4.76	0.47**	NNR	1	3.03	0.20
CD	36		1	2.38	0.39	GR	1	3.03	0.39**
DA	70		0	0	0.20		0	0	0.31
DB	58.3		1	2.38	0.24		0	0	0.24
DC	36	GR/NNR	3	7.14	0.39	GR/NNR	3	9.09	0.39**
ABC	66	GR/NNR	3	7.14	0.32	NNR	1	3.03	0.26
ABD	94.3		0	0	0.22	GR	0	0	0.27
ACB	88.3		0	0	0.24		0	0	0.19
ACD	94.3		0	0	0.22	GR	1	3.03	0.27
ADB	95.3		0	0	0.22		0	0	0.26
ADC	106		0	0	0.20		0	0	0.24
BAC	94.3		0	0	0.22		0	0	0.18
BAD	106		0	0	0.20		0	0	0.24
BCA	88.3		0	0	0.24		0	0	0.19
BCD	66	GR/NNR	8	19.05	0.32	GR/NNR	1	3.03	0.26
BDA	128.3		0	0	0.16		0	0	0.19
BDC	94.3		0	0	0.22		0	0	0.18
CAB	94.3		0	0	0.22		0	0	0.18
CAD	128.3		0	0	0.16		0	0	0.19
CBA	66	GR/NNR	4	9.52	0.32	GR/NNR	9	27.27	0.26
CBD	88.3		0	0	0.24		0	0	0.19
CDA	106		0	0	0.20		0	0	0.24
CDB	94.3		0	0	0.22		1	3.03	0.18
DAB	106		0	0	0.20		0	0	0.24
DAC	128.3		0	0	0.16		0	0	0.19
DBA	94.3		0	0	0.22	GR	1	3.03	0.27
DBC	88.3		0	0	0.24		0	0	0.19
DCA	94.3		0	0	0.22	GR	0	0	0.27
DCB	66	GR/NNR	5	11.9	0.32	NNR	5	15.15	0.26
ABCD	102	GR/NNR/CH	2	4.76	0.27	GR/NNR/CH	1	3.03	0.27
ABDC	130.3		0	0	0.21		0	0	0.21
ACBD	146.6		0	0	0.19		0	0	0.19
ACDB	152.6		0	0	0.18		0	0	0.18
ADBC	158.3		0	0	0.18		0	0	0.18
ADCB	136		0	0	0.21		0	0	0.20
BACD	130.3	GR/NNR	0	0	0.21	GR/NNR	0	0	0.21
BADC	142		0	0	0.20		0	0	0.20
BCAD	158.3		0	0	0.18		0	0	0.18
BCDA	136	CH	0	0	0.21	CH	0	0	0.21
BDAC	186.6		0	0	0.15		0	0	0.15
BDCA	152.6		0	0	0.18		0	0	0.18
CABD	152.6		0	0	0.18		1	3.03	0.18
CADB	186.6		0	0	0.15		0	0	0.15
CBAD	136		0	0	0.21		0	0	0.20
CBDA	158.3		0	0	0.18		0	0	0.18
CDAB	142	CH	0	0	0.20	CH	0	0	0.20
CDBA	130.3	GR/NNR	0	0	0.21	GR/NNR	0	0	0.21
DABC	136	CH	0	0	0.21	CH	0	0	0.20
DACB	158.3		0	0	0.18		0	0	0.18
DBAC	152.6		0	0	0.18		0	0	0.18
DBCA	146.6		0	0	0.19		0	0	0.19
DCAB	130.3		0	0	0.21		0	0	0.21
DCBA	102	GR/NNR	5	11.9	0.27	GR/NNR	4	12.12	0.27

GR = Gravity Rule; NNR = Nearest Neighbor Rule; CH = Convex Hull; Line in blanks = Other paths (non-heuristic strategies); F = Frequency of use; PI = Profitability Index; **Highest profitability indices in each condition

We performed Pearson’s chi-square test in each experimental condition to verify if the number of possible (expected proportion) and used routes (observed proportion) were the same for each strategy (heuristic strategy and other paths). To determine whether routes consistent exclusively with GR or NNR strategies, as well as those incorporating both GR/NNR strategies in Condition II, were used more frequently than expected by chance, we conducted binomial tests. We determined the chance probabilities for GR, NNR and GR/NNR strategies as 0.29, 0.19 and 0.33, respectively (please keep in mind that in our chance probabilities, we took into account both the CH strategies and the combined GR/NNR/CH strategies, please see Table 1).The data were analyzed in R, version 4.0.4 (R Core Team 2020) and the results were considered significant when the p-value was ≤ 0.05.

## Results

**Fig. 4:**
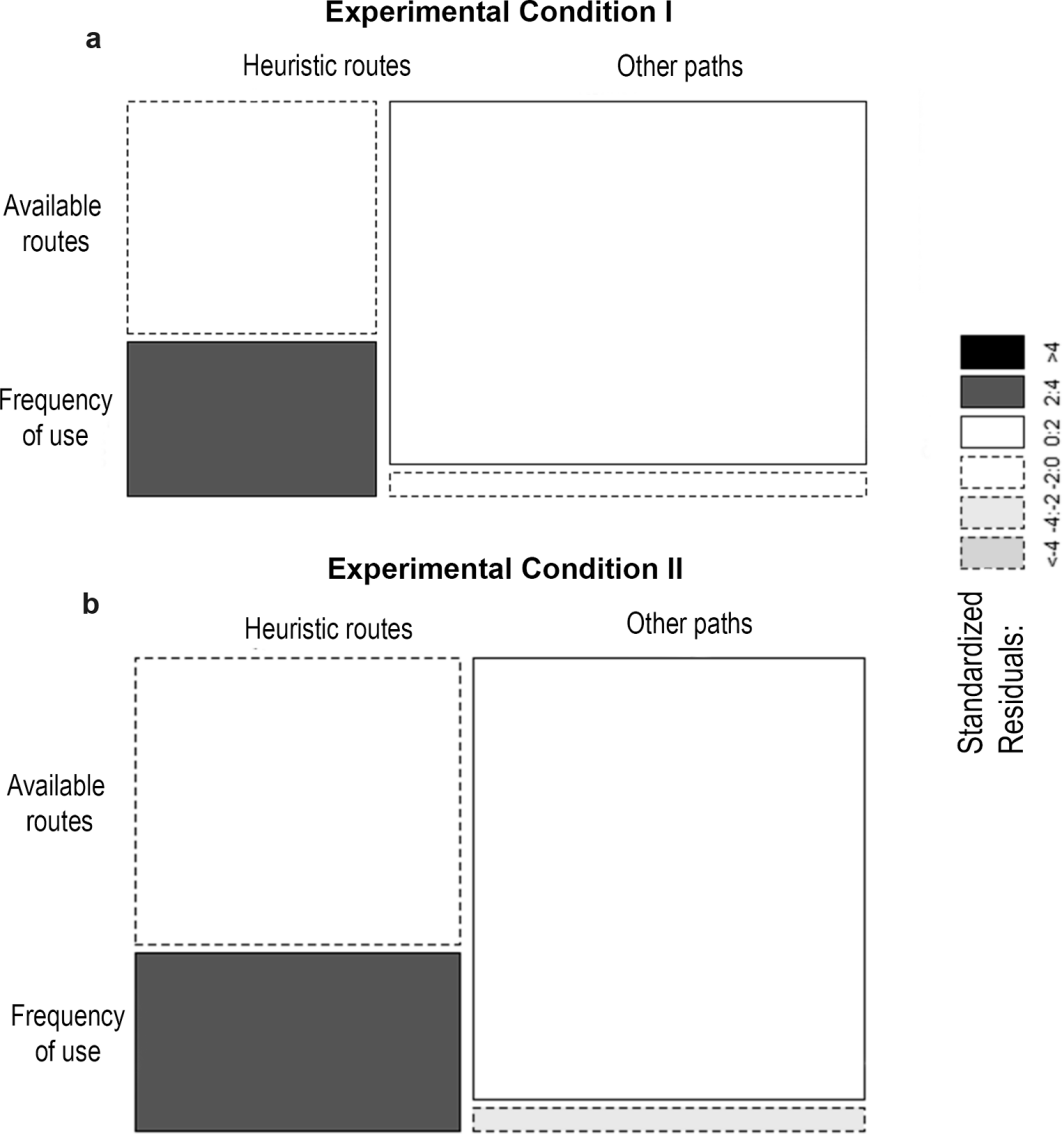
Mosaic plot of the routes used in both experimental conditions by the common marmosets. The cell width relative to the axis represents the proportional contribution of each variable to the total. The colors represent the level of standardized residuals (Pearson) for each combination of variables, with black representing the use of strategies more often than expected at random, and gray representing the use of strategies less often than expected at random

In total 99 experimental sessions were carried out, of which 75 were considered routes. In experimental condition I, where the food distribution was homogeneous between the platforms, the common marmosets performed 42 valid sessions, using a total of 14 different routes. Of these routes, the most frequently used were those with three platforms (48%, *n* = 20), followed by those with two platforms (36%, *n* = 15), with BCD being the most used route (19%, *n* = 8) (for more details see Table 1 and Online Resource 1). Routes consisting of four platforms were chosen by less than 17% of the animals (*n* = 7). In addition, routes including platforms B (*n* = 38) and C (*n* = 38) were used up to twice as often as routes including platforms A (*n* = 17) and D (*n* = 25). Furthermore, in this experimental condition, marmosets displayed a preference to use significantly more paths consistent with heuristic strategies, since we observed that they adopted these routes more than expected by chance (χ2 = 10.591, df = 1, *p* = 0.001) (Fig. 4).

In experimental condition II, characterized by a heterogeneous food distribution between platforms, the common marmosets performed 33 valid sessions, covering 15 different routes. Of these routes, those consisting of three platforms were chosen most frequently (58%, *n* = 19), with CBA being the most used route (28%, *n* = 9) (Table 1 and Online Resource 1); routes with two and four platforms were chosen less frequently (respectively, 24%, *n* = 8; 18%, *n* = 6). Similarly to what was observed in experimental condition I, routes including platforms B (*n* = 28) and C (*n* = 31) were visited about 1.5 times more often than routes including platforms A (*n* = 19) and D (*n* = 19). Therefore, once more, common marmosets chose their paths consistent with heuristic strategies more often than expected by chance (χ2 = 10.925, df = 1, *p* = 0.0009) (Fig. 4).

**Fig. 5:**
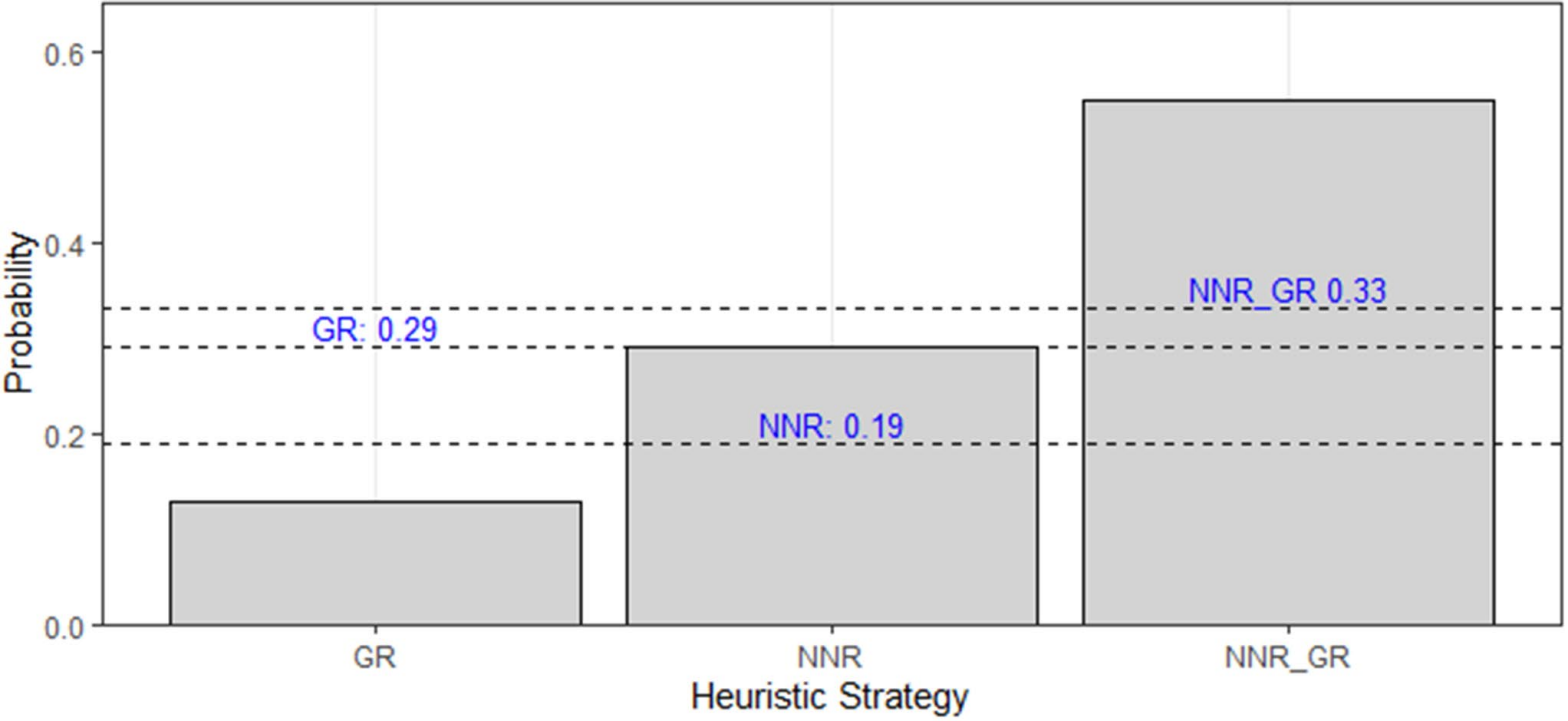
Probability distribution of the heuristic strategies: “GR”, “NNR” and “GR/NNR” during Condition II. The observed probabilities are represented by the gray bars, and the expected probabilities are indicated by the dashed black lines. The blue text above each line displays the respective expected probabilities for each heuristic strategy

Finally, our results showed that common marmosets do not use routes solely consistent with GR or NNR strategies more than expected by chance during Condition II. We found that animals used routes consistent with the GR strategy less than expected by chance (exact binomial test: *p* = 0.049, n_total_ = 31, n_observed_ = 4). In contrast, we found no significant statistical difference between the observed and expected number in the routes used by common marmosets that were only consistent with NNR strategy (exact binomial test: *p* = 0.16, n_total_ = 31, n_observed_ = 9). Moreover, we found that marmosets use routes that are both consistent with GR and NNR strategies more than expected by chance (exact binomial test: *p* = 0.01, n_total_ = 31, n_observed_ = 17) (Fig. 5).

## Discussion

In this study, we aimed to test if wild common marmosets use routes consistent with heuristic strategies to efficiently navigate through multiple feeding sites in a large-scale space. According to our first prediction, the animals used routes consistent with heuristic strategies to choose their navigation paths more than expected by chance. Our results are in line with those described by Teichroeb (2015), Teichroeb and Smeltzer (2018), Kumpan et al. (2019) and Joyce et al. (2023), who also reported the use of heuristic strategies by vervet monkeys (*Chlorocebus pygerythrus*) and Japanese macaques (*Macaca fuscata*) to solve multi-destination routing problems. However, our second prediction was not supported, as GR strategies alone were not used more frequently than expected by chance during Condition II. In our experiment, common marmosets only chose the most profitable routes (GR) when these coincided with the shortest routes (NNR). These findings contrast with previous results from Jason (2007) that documented route selection based on profitability by the black capuchin monkeys (*Sapajus nigritus*, formerly *Cebus apella nigritus*), another Neotropical primate. This author observed that these primates take detours to reach more distant feeding sites only when the amount of food available there outweighs the energy expenditure required to travel to those locations. Nonetheless, our results bring preliminary and relevant insights into the common marmoset’s navigation movements when foraging in large-scale space, suggesting that marmosets can be flexible and have the ability to integrate multiple heuristic strategies to choose the best route to travel.

Navigation routes require the ability to rely on spatial memory and the capacity to anticipate the feeding sites they will encounter (i.e., the ability to plan). Although this ability was considered exclusively human (Clayton et al. 2003), in the last few years a number of studies have found that non-human primates can retain information from past encounters (episodic memory) to anticipate future encounters (planning) (e.g.: Chimpanzees, *Pan troglodytes*, Janmaat et al. 2013; Ban et al. 2014; Green et al. 2019; chacma baboons, *Papio ursinus*, Noser and Byrne 2007; gibbons, *Hylobates lar*, Asensio et al. 2011; spider monkeys, *Ateles geoffroyi yucatanensis*, Valero and Byrne 2007; capuchin monkeys, *Sapajus nigritus*, Janson 2007; for a review see Trapanese et al. 2018). A more recent study, conducted by Abreu et al. (2021) on wild common marmosets’ travel routes showed that this species relies on a route-based mental map to navigate their environment and revisited the same feeding sites over the course of nine weeks. Additionally, the authors suggested the use of episodic memory by this species in small-scale space (Abreu et al. 2020).

For common marmosets, whose diet includes resources characterized by both patchy (e.g., fruits, flowers), predictable and unpredictable resources (e.g., exudates and insects; Souto et al. 2007; Abreu et al. 2016; Schiel and Souto 2017), the ability to use heuristic strategies, and the capacity to integrate multiple strategies to select their routes when foraging has several advantages. For example, by being capable of selecting a route considering different aspects of the environment, the animal can assess the surrounding environment and dynamically adjust their movement decisions according to the environmental conditions, resources eaten, and other ecological and social factors that have been shown to influence movement decisions (e.g., Cunningham and Janson 2007/2013; Hopkins 2016; Kumpan et al. 2022; Teichroeb et al. 2023). In our experiment, however, we also observed that the pattern of visiting all four platforms was the least utilized in both experimental conditions. This contrasts with the pattern observed in small-scale spaces in other primate species, such as lemurs and vervet monkeys (e.g., Teichroeb 2015; Teichroeb and Vining 2019). This difference is likely a result of the animals being potentially distracted by other resources or events, such as predation, which are less common in small-scale-space field experiments (Garber and Dolins 2010). Navigating over a large-scale space requires the animal to resort to different strategies to make travel costs more efficient by prioritizing the most relevant resources in its home range (Garber and Dolins 2010; Asensio et al. 2011). For example, in our experiment platforms B and C (the two closer platforms with less food available) were visited more often in both experimental conditions. Prioritizing the use of certain feeding sites over others can be important to exploit more efficiently the resources available in the environment, minimizing the traveled distance and obtaining as much food as possible during a limited time frame. The capacity to make faster and profitable decisions on where to navigate is especially important in the Caatinga environment due to the possible constraints and limitations that the environment imposes on marmoset navigation (Abreu et al. 2021).

Especially with large-scale space navigation, the ability to plan becomes even more essential. Since large-scale space resources are not visible to the foraging individual, animals need to resort to their spatial memory to guide their navigation and improve the chances of finding resources (Garber and Dolins 2010). The relationship between the use of spatial memory and increased efficiency in selecting routes in large-scale space has already been documented in spider monkeys (*Ateles geoffroyi yucatanensis*) by Valero and Byrne (2007). Like common marmosets, these primates also have a diet characterized by foods that are patchily distributed throughout the environment. As referred by Teichroeb and Vining (2019), this endorses the hypothesis that successfully exploring stationary resources like fruit implies the use of foraging strategies related to spatial memory to select faster and more efficient routes.

Furthermore, our results regarding the use of GR strategy in the scenario with different quantities of food were not expected and contradicts the numerical ability of common marmosets highlighted in Stevens et al.’s (2007) study. The study shows that marmosets can distinguish between two amounts of food, supporting their ability to quantitatively assess available resources in the environment before exploiting them. Moreover, our results also did not support the sole use of routes consistent with the NNR strategy. Instead, they showed that common marmosets prefer to use routes that were mainly consistent with both strategies, GR/NNR. One possible explanation and limitation, which cannot be ruled out, is the limited number of routes solely consistent with the GR strategy. While this could influence the outcome, it does not fully explain the other results encountered. Thus, another plausible explanation is that, similar to their capacity to adapt and/or adjust some traits in the Caatinga environment (resting behavior: De la Fuente et al. 2014; feeding behavior: Abreu et al. 2016; social structure and morphology: Garber et al. 2019; physiology: Garber et al. 2020), marmosets can also flexibly use and switch between two different heuristic strategies, considering both the quantity of food available and the distance between platforms. Using this “integrated strategy” would enable common marmosets to optimize their navigation by exploiting routes with a higher profitability index and shorter distances traveled (Lihoreau et al. 2012b; Reynolds et al. 2013; Teichroeb et al. 2023), a crucial aspect for groups of Caatinga common marmosets when traveling in large-scale space. Moreover, in addition to predation events or intragroup interactions, Caatinga common marmosets must cope with extreme temperatures during the day, which has already been suggested to influence navigation decisions (Abreu et al., in prep.) and other behaviors (De la Fuente et al. 2014; Abreu et al. 2016). Therefore, choosing the routes that are consistent with both strategies may lead them to make quicker, safer and less effort movement decisions in an environment that is extremely challenging for mammals.

Overall, our study provides a better understanding of the importance of spatial cognitive skills in the behavioral flexibility of wild primates when foraging in an environment characterized by periodic variation in resource availability, such as the Caatinga (Salimon and Anderson 2018). Due to the increased distance and decreased visibility between feeding sites on a large-scale space (Garber and Dolins 2010), using heuristic decisions is critical in choosing the most efficient routes to maximize energy gain (Teichroeb et al. 2023). However, our results should be regarded with caution, as factors that we have not analyzed (e.g., hierarchy, sex, age, etc.) could potentially affect the individual choices of these primates while navigating on a large-scale space (as documented in vervet monkeys by Teichroeb and Aguado 2016). Nevertheless, it is important to note that the arrangement of the platforms did not allow us to adequately test or make comprehensive comparisons between these and other strategies. Future field experiments in large-scale space, based on a similar paradigm with variations, may offer insights into specific heuristic strategies at play during navigation in Caatinga common marmosets and other primate species. Despite these limitations, our study brings important insights into common marmosets’ spatial cognition by showing that this species can use heuristic decisions to select the most efficient routes available across multiple sites distributed over large-scale space.

## Electronic supplementary material

Below is the link to the electronic supplementary material.

Supplementary Material 1

## Data Availability

Not applicable.
