# Supplementary material for: Choosing the best way: how wild common marmosets travel to efficiently exploit resources
Source: Anim Cogn. 2024 Mar 2;27(1):20. doi: 10.1007/s10071-024-01864-8 (PMC10907437; doi:10.1007/s10071-024-01864-8)
Supplement: Supplementary file 1 — Supplementary Material 1 [file 10071_2024_1864_MOESM1_ESM.docx]

# Choosing the best way: how wild common marmosets travel to efficiently exploit resources

Animal Cognition

Dêverton Plácido Xavier 1 Filipa Abreu 1* Antonio Souto 2 Nicola Schiel 1

1 Laboratory of Theoretical and Applied Ethology, Department of Biology, Federal Rural University of

Pernambuco, Recife, Brazil

E-mail: filipaabreu88@gmail.com

**Table S1** Chi-square table describing the possible and used routes by marmosets consistent either with heuristic or non-heuristic strategies (other paths)

|  | Possible Routes | Used Routes |
| --- | --- | --- |
|  | Experimental Condition I | |
| Heuristic | 15 | 10 |
| Other Paths | 45 | 3 |
|  | Experimental Condition II | |
| Heuristic | 21 | 13 |
| Other Paths | 39 | 2 |
